# Progress in Bacteriology

**Published:** 1900-04-07

**Authors:** 


### Progress in Bacteriology.

**Scarlet Fever.**—Class[1] has obtained from the scales and throat secretions of scarlet fever patients a large diplococcus or tetracoccus. It is stained by anilin dyes and decolorised by Gram. Blood serum is the most favourable medium for growth. Injected into swine it excites a febrile attack and exfoliation resembling scarlet fever, and is found in the blood and skin of the inoculated animal. Caddy and Cooks'[2] experiments in India with a streptococcus isolated from a case of scarlet fever have drawn attention again to Klein's streptococcus scarlatinæ, but the opinion is generally held that, whilst streptococci may induce a septic state simulating scarlet fever, there is no true connection between the two. Eddington's description of the Bacillus scarlatinæ has not been confirmed. Curtois[3] supports the opinion that the specific organism of the disease is a streptococcus analogous to that of erysipelas. He found streptococci in the urine of scarlet fever patients on 42 occasions out of 97 examined. Albuminous urines contained streptococci more frequently than non-albuminous. The cocci may be found in the heart's blood after death. It is probably a variety of streptococcus distinct from S. pyogenes and erysipelatosus.

**Diphtheria.**—Further proof of the identity of the pseudo-diphtheria (Hoffman) bacillus with the true Klebs-Löffler bacillus is furnished by Salter.[4] It was found that the pseudo-bacillus, whilst non-pathogenic to guinea pigs and large birds, such as the thrush and blackbird, was pathogenic to small birds of the finch variety. By successive passages through small birds and then through larger ones, Salter has been able to raise the virulence of Hoffman's bacillus, and to render it pathogenic to rabbits and guinea-pigs. In the process of conversion the type of organism is completely altered, and although its toxin was not exactly identical with that of the Klebs-Löffler bacillus, yet it possessed in a very high degree the quality of neutralising diphtheria antitoxin. In this connection it is interesting to note that Macfadyen and Hewlett[5] have recently described in the throats of diseased and healthy pigeons an organism which resembles the Klebs-Löffler bacillus in morphological, cultural, and staining peculiarities, and differing only slightly in pathogenic and toxic properties. Hewlett,[6] after an extended trial, is of opinion the reaction of the Klebs-Löffler bacillus to Neisser's stain is a constant and reliable feature serving to distinguish the organism from other types, such as the pseudo-bacillus, xerosis bacillus, &c. In Neisser's stain two solutions are employed: (1) One gramme of methylene blue dissolved in 20cc. of 96 per cent. alcohol, mixed with 950cc. of distilled water and 50cc. of glacial acetic acid; and (2) two grammes of vesuvin are dissolved in 1,000cc. of boiling distilled water, and the solution cooled and filtered. Cover-glass preparations should be made from serum cultures (not agar) not more than 24 hours old, and stained for five seconds in the blue, rinsed in water, and counterstained for 10 seconds in the brown. The true bacillus appears as a slender longish rod, stained brown, and containing granules of a deep blue or inky tint.

**Whooping-cough.**—Koplik,[7] examining the sputum pellets in this disease previous to the mucopurulent stage, confirms Afanassjew's statement as to the presence of a distinctive bacillus. The bacillus may be seen in films of the sputum stained with methylene blue, both within and outside the epithelial cells, in zoogloea masses. It grows best on hydrocele fluid, but may be cultivated on most media. It is a delicate, short bacillus, measuring $\cdot 8\mu$. to $1\cdot 7\mu$. long and $\cdot 3\mu$. to $\cdot 4\mu$. wide, staining irregularly, and apt to undergo involution. No spores can be detected. That the bacillus is the direct exciting cause of whooping-cough cannot as yet be maintained, for no convulsive symptoms followed the injection of a culture into animals.

**Eclampsia.**—Lermovitsch[8] found a definite bacillus in the blood of 44 cases of puerperal convulsions. The organism described is round or oval in shape (planococci), and bears flagellæ. Growth takes place on gelatine and agar, and in broth. The blood is best examined during a convulsion. The organism, when injected into guinea-pigs and rabbits, excited hæmorrhagic endometris, anæmia, and tetanic spasms.

**Yellow Fever.**[9]—The causal relationship of the Bacillus icteroides to yellow fever has been confirmed by the Commission appointed by the Marine Hospital Service of Washington. The bacillus was obtained from 83 per cent. of the cases examined, and was not present in diseases other than yellow fever. The Bacillus icteroides is naturally infectious to such animals as rats and dogs, infection taking place not by the alimentary but by the respiratory tract. The disease is essentially a lung affection. Whilst drying and cold do not markedly affect its vitality, the bacillus is easily killed by weak antiseptics, or by sunlight. Sanarelli[10] thinks that the specific pathogenicity of the Bacillus icteroides can no longer be doubted. He mentions that the serum from 93 per cent. of patients with yellow fever causes agglutination of B. icteroides, and the introduction of the organism into the system of the lower animals is followed by a well-marked acute fatty degeneration of the liver.

**Bacteria and Digestion.**—Nuttall and Thierfelder found that young animals removed from their mothers by Cæsarian section, and kept and fed under aseptic conditions, did not put on weight so rapidly as did similar animals living and fed under natural conditions. Schottelius[11] has been able to hatch and rear the chickens under aseptic conditions, and to feed them on aseptic food. Up to the twelfth day their weight had increased about 25 per cent., but then stopped. Control chicks had in the same time increased 140 per cent., and in 17 days 250 per cent. Although the excreta of the experimental chicks were sterile, there was after the fourth day an enzyme present capable of digesting gelatine very slowly. Probably the enzymes are incapable of exerting full power until aided by bacteria, which appear normally in the chicks' excreta in 36 hours, up to which period no increase in body weight occurs.

Many of the organisms inhabiting the digestive tract are normally present in the mouth; amongst these we may mention streptococcus brevis, B. fluorescens, liquefaciens motiles, and a red pigment forming motile bacillus. Staphylococcus albus, citreus, and aureus are generally present, and on potato gelatin there may be separated the motile Spirillum sputugenum, which liquifies gelatine, and gives the cholera red re-action.[12] Another motile organism is the B. maximus buccalis or Leptothrix buccalis maxima of Miller. Most of these microbes grow best on an alkaline medium.

Although neutral in reaction, bile is generally sterile. Fraenkel and Krause[13] examined the contents of 130 gall-bladders, and in only 25 cases were organisms detected. These 25 cases included 11 of cholelithiasis, four of peritonitis, three following abdominal operations, two of tuberculosis, and four of acute fever. The B. coli and cocci were the organisms most commonly found. Experiments showed that B. typhosus, B. coli, B. pyocyaneus, the cholera vibrio, and staphylococci could live for prolonged periods in bile, but streptococci, B. diphtheria, and pneumococcus perished much more quickly.

[1] Sanitarian, Aug., 1899. [2] Indian Med. Gaz., Aug., 1899. [3] Thèse de Paris, 1899. [4] Trans. Jenner Inst., second series. [5] Brit. Med. Jour., Nov. 11, 1899. [6] Trans. Jenner Inst., second series. [7] Brit. Med. Jour., Oct. 16, 1899. [8] Centrab. f. Gynak., No. 46, 1899. [9] Brit. Med. Jour., Feb. 10, 1900. [10] New York Med. News, Dec. 9, 1899. [11] Archiv. fur Hyg., Bd. xxxiv., 1899. [12] Trans. Odont. Soc., xxx., No. 8, p. 171. [13] Zeit. fur Hyg., xxxii., 1899.

